# Metabarcoding Unveils Seasonal Soil Microbiota Shifts and Their Influence on *Boletus edulis* and *Boletus reticulatus* Mycelium in *Quercus robur* Stands

**DOI:** 10.3390/microorganisms13092196

**Published:** 2025-09-19

**Authors:** Serena Santolamazza-Carbone, Laura Iglesias-Bernabé, Elena Benito-Rueda, Esther Barreal, Pedro Pablo Gallego

**Affiliations:** Applied Plant & Soil Biology, Plant Biology and Soil Science Department, Biology Faculty, University of Vigo, E-36310 Vigo, Spain; lauib@uvigo.gal (L.I.-B.); rueda@uvigo.es (E.B.-R.); edesther@uvigo.es (E.B.); pgallego@uvigo.es (P.P.G.)

**Keywords:** extraradical mycelium, microbiota diversity, pedunculate oak, qPCR, seasonal dynamics, soil microbial communities, mycorrhizosphere, high-throughput sequencing analysis, fungus–bacteria interactions

## Abstract

Forest ecosystems undergo seasonal shifts in bacterial and fungal communities, but little is known about the specific microbiota associated with *Quercus robur*–*Boletus edulis* systems. This study represents the first examination of seasonal changes in soil microbiota in pedunculate oak habitats in Galicia (NW Spain) and their relationship with *Boletus edulis* and *Boletus reticulatus* mycelium prevalence and concentration. Soil microbiota richness, diversity, and composition, as well as seasonal variation in *Boletus* mycelium, were assessed using DNA metabarcoding and qPCR, respectively. Sampling was conducted in autumn at two 30–40-year-old *Q. robur* stands. Bacterial communities were dominated by *Acidobacteria* (34%) and *Proteobacteria* (33%), with *Acidobacterium* (12%), *Paludibaculum* (9%), and *Edaphobacter* (7%) identified as most abundant. Fungal communities were primarily Basidiomycota (93%), led by *Russula* (46%). For both bacteria and fungi, the highest OTU richness was observed in September, followed by a significant decrease in October and a partial recovery in November. *Boletus* species were found to exhibit positive correlations with specific bacteria (e.g., *Massilia*, *Rhizobium*) and fungi (e.g., *Amanita*, *Clavaria*, *Inocybe*, *Scleroderma*, *Suillus* and *Mortierella*), suggesting a potential influence of these microbes on mycelium development. This study provides novel insights into the seasonal dynamics of soil microbiota and their potential role in *Boletus* ecology, thereby advancing understanding of host–microbe interactions in temperate forests.

## 1. Introduction

Atlantic European oak forests, dominated by *Quercus robur* and *Q. petraea*, extend from northern Portugal to Norway, encompassing the UK and Ireland. These woodlands thrive in a wet, oceanic climate and support rich understory vegetation [1]. In Galicia (NW Spain), *Q. robur* constitutes the dominant forest species, covering approximately 124,800 ha (26% of forested land), with an additional 248,200 ha of mixed *Q. robur*–*Q. petraea* stands [2]. Despite being the region’s climax vegetation, these oak forests have suffered extensive deforestation over centuries, largely for shipbuilding and railway construction [3,4]. Consequently, much of the landscape is now dominated by pine and eucalypt plantations. Nevertheless, ecologically valuable oak stands persist, and conservation efforts are currently underway for their preservation and restoration [5,6].

*Quercus robur* is known to form symbiotic ectomycorrhizal relationships with over 230 identified fungal species in Britain [7]. The composition of these fungal communities has been investigated using several approaches. Qualitative studies of the fungal assemblage have been conducted through macrofungal surveys in Ireland [8], and Sweden [9]. More recently, mycorrhizal root tips have been collected and analyzed by molecular techniques in Poland [10], Spain [11,12], and Belgium [13]. This approach has allowed for a direct assessment of the fungal partners associated with *Q. robur* roots. However, current applications of DNA metabarcoding to *Q. robur* soil fungal communities have mainly targeted the detection of fungal pathogens [12,14,15].

The *Boletus edulis* complex, which includes *B. edulis* Bull.: Fr. sensu stricto, *B. aereus* Bull.: Fr., *B. pinophilus* Pilat et Dermek, and *B. reticulatus* Schaeff., represents a group of highly valuable edible ectomycorrhizal fungi [16] hosted by *Quercus* species. These mushrooms exhibit a broad geographic distribution across Eurasia and North America and have also been introduced into several Southern Hemisphere countries, including South Africa and New Zealand [16].

Wild edible fungi, like other valuable non-timber products, comprise a high-value resource in forest areas. All members of the *B. edulis* complex have excellent culinary qualities, which make them the most widely collected species globally [17].

The ectomycorrhizal relationships formed by *B. edulis* with various tree communities have been documented in several European countries. Italian studies have reported associations with important forest tree species such as *Quercus cerris* L., *Q. rubra* L., and *Castanea sativa* Mill. [18], *Abies alba* Mill., *Picea abies* L., *Pinus nigra* J.F. Arnold, *Larix decidua* Mill. and *Fagus sylvatica* L. [19], *C. sativa* [20], and *Q. cerris*, *C. sativa* and *F. sylvatica* [21]. In contrast, research in Spain has only reported *B. edulis* in association with *Pinus sylvestris* L. [22,23,24], *Cistus ladanifer* L. [25,26], and *Castanea x coudercii* [27]. Compared to *B. edulis*, studies on *B. reticulatus* are scarce. The existing literature on *B. reticulatus* primarily focuses on heavy metal accumulation in the sporocarps [28,29], in vitro culture [30], and species-specific genes to prevent food adulteration [31].

Recent studies suggest that soil bacterial communities may also influence *Boletus* sp. mycelium development, extending beyond the established ectomycorrhizal relationship with host trees. For instance, Peintner et al. [20] observed a lack of correlation between extramatrical mycorrhizal fungal abundance and sporocarp production in *B. edulis*, suggesting that factors beyond fungal biomass play a role. Supporting this hypothesis, Mediavilla et al. [26] have used indicator species analysis (ISA) to identify bacterial genera potentially associated with high *B. edulis* sporocarp productivity. Their results highlight bacteria from genera such as *Archangium*, *Azospirillum*, *Conexibacter*, *Ferruginibacter*, *Gemmatimonas*, *Opitutus*, *Mucilaginibacter*, *Stella*, and *Terriglobus* as potential indicators.

Of particular interest within the soil microbiota are mycorrhiza helper bacteria (MHB), which are known to promote mycorrhizal fungi growth and enhance the formation and function of symbiotic associations between fungi and plant roots. These bacteria can reduce fungal stress, stimulate fungal development, and increase root–fungus contact points, thereby supporting healthier and more effective mycorrhizal relationships [32,33,34]. In vitro studies have demonstrated promising results with MHBs for *B. edulis*. Significantly increased mycorrhization of *B. edulis* with *Cistus ladanifer* has been reported by Mediavilla et al. [35] when co-inoculated with the *Pseudomonas fluorescens*. Similarly, enhanced mycorrhization of *B. edulis* with *Pinus thunbergii* Parl. has been observed by Wu et al. [36] in the presence of *Bacillus cereus*.

Metabarcoding and metagenomics are two prominent approaches for studying soil microbial communities. Metabarcoding involves identifying community composition by analyzing sequences of specific barcode marker genes [37]. Conversely, metagenomics focuses on the sequencing and analyzing of the collective genomes of all microorganisms within the community. While metagenomics offers a more comprehensive view, DNA metabarcoding has emerged as a valuable tool for investigating *B. edulis* microbiota. Currently, only four studies have analyzed the microbial community associated with *B. edulis* by using DNA metabarcoding. Mediavilla et al. [26] have investigated the effect of forest fire prevention on bacterial communities in *Cistus ladanifer* scrublands, identifying bacterial taxa potentially influencing *B. edulis* sporocarp production and mycelial biomass. Qin et al. [38] have examined bacterial diversity in the soil surrounding *B. edulis* mycelium, providing insights into the local bacterial assemblage. Koskinen et al. [39] have developed a protocol for extracting amplifiable DNA from fungal samples and applied it to investigate bacterial, fungal and arthropods communities within *Picea* stands. Santolamazza-Carbone et al. [27] have assessed positive correlations between *B. edulis* mycelium concentration and soil microbiota in chestnut orchards of varying ages.

Forest ecosystems exhibit dynamic shifts in bacterial and fungal communities throughout the year. These variations are likely to correspond to seasonal changes in plant inputs, organic matter accumulation, temperature, and rainfall patterns [40]. At present, no information is available concerning the specific bacterial and fungal communities associated with the rhizosphere of the *Q. robur*-*Boletus edulis* complex. Furthermore, the potential influence of the soil microbiota on the abundance of *B. edulis* and *B. reticulatus* mycelia in this habitat remains poorly understood.

This study examines whether seasonal shifts in soil microbiota within *Quercus robur* habitats influence the prevalence and abundance of *Boletus edulis* and *B. reticulatus* mycelia. To address this hypothesis, three main objectives are pursued: (1) to assess soil microbiota richness, diversity and assemblage in pedunculate oak stands across autumn, evaluating potential variations; (2) to investigate changes in the prevalence and concentration of *B. edulis* and *B. reticulatus* mycelia between sampling months, considering the influence of local climatic parameters; and (3) to identify key taxa of soil bacteria and fungi involved in significant correlations with the mycelium concentration of *B. edulis* and *B. reticulatus*.

## 2. Materials and Methods

### 2.1. Study Site, Soil Characteristics and Climate

The study site is a mature pedunculate oak (*Q. robur*) stand located in Pontevedra province, Galicia, NW Spain. Granites and low-grade metamorphic rocks dominate the country’s diverse geology. Pontevedra shares this composition, resulting in predominantly Leptosols, Umbrisols, Cambisols, and Regosols. Galicia is characterized by a humid Atlantic climate with a mild mean annual temperature (14.8 °C) and low thermal amplitude (11 °C). Annual precipitation is high (1613 mm), with significant seasonal variability, the autumn period being the rainiest season and the months of July and August exhibiting the lowest precipitation (44 and 56 mm, respectively), although no dry month was observed throughout the year (www.meteogalicia.gal; accessed on 1 December 2020). The high precipitation of the region results in markedly acidic soils, containing abundant organic matter and low levels of nutrients [41,42].

### 2.2. Experimental Design

Two mature pedunculate oak (*Q. robur*) stands (30–40 years old), named A and B, were selected in Bora (Pontevedra province, 42°25′56.5″ N–8°34′41″ W). Both stands are surrounded by pastures, fruit orchards, and mature chestnuts and are located approximately 800 m apart, sharing similar climatic conditions and soil characteristics. Within each stand, a 100 m^2^ area was designated for soil sampling.

To assess the main soil physical and chemical properties, soil samplings were carried out in September 2020. Three soil samples were collected at a depth of 0–5 cm using a soil corer (5 × 5 cm) for randomly distributed locations within each 100 m^2^ stand for bulk density determination. An additional set of three soil samples per stand was collected at a depth of 20 cm for analysis of other soil properties.

Most of the soil characteristics were determined according to international standards [43]: sand, silt, and clay%; bulk density (BD, kg m^−3^), base saturation (V%), total porosity (Pt,%), pH, exchange cations (Ca^2+^, Mg^2+^, K^+^, Na^+^, Al^3+^) and cation exchange capacity (CEC, cmol(+)kg^−1^). Total C and N were determined with an elemental analyzer and available P by the Olsen method [44].

Climatic data (mean, maximum and minimum temperatures (T, °C), air relative humidity (RH,%), precipitation (P, mm), potential evapotranspiration (PET, mm), and water balance (WB, mm) for September, October, and November 2020 were obtained from the closest meteorological station (42°23′28″ N–8°40′03″ W), located 4 km apart from the sampling sites (http://www.meteogalicia.gal; accessed on 1 December 2020). Due to the relatively short distance and similar landscape features, data from this station were considered representative of the microclimatic conditions at the study site.

The presence of *B. edulis* complex sporocarps was confirmed within the experimental area during the autumn season. Notably, October yielded the highest number of fruiting bodies (17) with an even distribution across both plots (8 in plot A and 9 in plot B). This observation, coupled with the general abundance of *Boletus* species in late autumn, aligned the selection of the sampling period with September, October, and November 2020 [27].

To investigate soil microbiota, a total of 30 soil samples were collected (2 experimental stands × 3 sampling months × 5 soil samples) using a 250 cm^3^ soil extractor (3.5 cm diameter and 20 cm deep). Within each plot, the five samples were distributed randomly across the 100 m^2^ area. Following the procedure described elsewhere [45], samples were collected near the corners and center of the stand, maintaining a maximum distance of 30 cm from the tree trunk.

The soil samples were individually introduced in marked plastics bags and transported to the laboratory, where they were stored at 4 °C and processed within 24–48 h. Soil DNA extractions were carried out with the DNeasy^®^ PowerSoil^®^ Kit (QIAGEN Group, Venlo, The Netherlands), from 0.25 g of soil per sample according to the manufacturer’s instruction. The extracted DNA was diluted in 100 µL of buffer solution and stored at −20 °C until used. For each combination of experimental stand (*n* = 2) and sampling month (*n* = 3), a bulk of 25 µL was prepared by pooling 5 µL of DNA extract from each of the five individual soil samples collected. This pooling strategy aimed to obtain sufficient DNA quantity and to represent the overall microbial community within each plot at each time point.

### 2.3. Metabarcoding Analysis for Bacterial and Fungal Identification

Following successful DNA extraction, the six samples (2 × 3 × 1 pooled sample) were sent to Macrogen laboratory (Macrogen Inc., Seoul, Republic of Korea, www.macrogen.com) for analysis of bacterial and fungal communities using DNA metabarcoding on the Illumina platform. Quality control measures were performed during DNA extraction, including spectrophotometric assessment of purity (A260/280 and A260/230 ratios) and agarose gel electrophoresis to confirm DNA integrity. Additionally, inhibitor presence was evaluated by dilution tests and confirmed to be negligible.

The targeted gene regions for identification were the 16S rRNA gene for bacteria and the ITS2 region for fungi. The following specific primers with Illumina adapters were used for PCR amplification during library preparation before sequencing. The primers Bakt-341F (CCTACGGGNGGCWGCAG) and Bakt-805R (GACTACHVGGGTATCTAATCC) were used to amplify the V3–V4 region of bacterial 16S rDNA [46]. The primer pair ITS3 (GCATCGATGAAGAACGCAGC) and ITS4 (TCCTCCGCTTATTGATATGC) were adopted to amplify the region of the internal transcriber spacer ITS2, which lies between the 5.8S and the 28S genes of fungal rDNA [47,48]. Two microliters of the genomic DNA (10 ng) were PCR amplified with 5× reaction buffer, 1 mM of dNTP mix, 500 nM each of the universal PCR primers, and Herculase II fusion DNA polymerase (Agilent Technologies, Santa Clara, CA, USA). The cycle condition for the first PCR was 3 min at 95 °C for heat activation, and 25 cycles of 30 s at 95 °C, 30 s at 55 °C and 30 s at 72 °C, followed by a 5-min final extension at 72 °C.

The first PCR product was purified with AMPure beads (Agencourt Bioscience, Beverly, MA, USA). Successively, 2 µL (20 ng) of first PCR product was PCR amplified for final library construction containing the index using NexteraXT Indexed Primer. The cycle condition for the second PCR was the same as of the first PCR, although only 10 cycles were performed. The PCR product was then purified with AMPure beads. The final purified product was then quantified using quantitative PCR (qPCR) according to the qPCR Quantification Protocol Guide (KAPA Library Quantificatoin kits for Illumina Sequecing platforms) and qualified using the Tape Station D1000 Screen Tape (Agilent Technologies, Waldbronn, Germany). Finally, samples were sequenced using the MiSeq™ platform (Illumina, San Diego, CA, USA).

Bioinformatic analyses were also performed by Macrogen laboratory. Raw sequencing data underwent quality control and processing using established bioinformatic tools. In brief, raw sequences were cleaned with the FASTP program (a FASTQ data pre-processing tool) [49], to remove adapter sequences and perform error-correction for areas where the two reads overlap. Paired-end reads were assembled using FLASH (v.1.2.11) [50] to generate longer, more accurate sequences. QIIME software (v 1.9.0) [51] was employed for OTU (Operational Taxonomic Units) analysis.

For bacteria, sequences shorter than 400 bp or longer than 500 bp were filtered out. Chimeric reads were removed with rDNATools PacBio by using RDP’s database (http://www.mothur.org/wiki/RDP_reference_files accessed on 14 August 2025). After, OTUs were clustered at a 97% sequence identity threshold using CD-HIT-OTU [52]. Taxonomic assignment of bacterial OTUs at genus level was performed using the NCBI 16S Microbial database with BLASTN (v 2.9.0) algorithm, requiring 97–100% identity for reliable identification.

Similar bioinformatic procedures were applied to the fungal data. Sequences shorter than 300 bp or longer than 500 bp were removed, and the UNITE Fungi database with UCLUST (v.1.2.22) algorithm was used for taxonomic identification. A more stringent identity threshold (99–100% with E-value = 0) was used for fungal species-level identification, whenever possible.

The raw sequencing data were deposited in NCBI GenBank (http://www.ncbi.nlm.nih.gov/ accessed on 14 August 2025) database as BioProject under the ID PRJN1277155. Also, this Targeted Locus Study project has been deposited at DDBJ/ENA/GenBank under the accession KJAV00000000. The version described in this paper is the first version, KJAV01000000.

### 2.4. Molecular Detection and Quantification of B. edulis and B. reticulatus Mycelium

The detection and quantification (mg mycelium/g soil) of *B. edulis* and *B. reticulatus* mycelia were carried out using quantitative PCR (qPCR). DNA samples were shipped to the AllGenetics laboratories (AllGenetics & Biology SL, La Coruña, Spain, www.allgenetics.eu) for analysis. The qPCR for *B. edulis* mycelium detection was performed using specific primers and probe designed by De la Varga et al. [48]. For *B. reticulatus* primers and probe were designed with Geneious 10.2.3 software (Biomatters Ltd., Auckland, New Zealand) [42]. Validation of the newly designed *B. reticulatus* primers and probe for qPCR was performed to ensure specificity and efficiency before their application in this study.

Five-fold dilution series (performed in triplicate) of known ITS copy numbers (from 6.16 × 10^6^ to 3.50 × 10^13^ in 100 µL of DNA solution) from an identified *B. edulis* sporocarp sample were used to establish the standard curves (one per plate; total of nine plates) and to assess the reaction efficiency. *Boletus edulis* mycelium quantification targeted the ITS genomic region using the primers FWD-Bedu (CTGTCGCCGGCAACGT) and RVS-Bedu (TGCACAGGTGGATAAGGAAACTAG), and TaqMan^®^ probe STQBedu (6FAM-CCCTTTCTCTTTCGTGGAACCTCCCC-BHQ1). The probe incorporated the dye 6-carboxy-fluorescein (6-FAM) at the 5′ and the Black Hole Quencher (BHQ1) at the 3′ ends. The qPCR reaction mixture (final volume of 20 μL) contained 10 μL of NZY qPCR Probe Master Mix ROX plus (NZYTech), 0.25 μM of the probe, 0.9 μM of each primer, 2 μL (20–30 ng) of template DNA, and ultrapure water (up to 20 μL). The thermal cycling conditions included an initial incubation at 95 °C for 10 min, followed by 40 cycles of denaturation (95 °C for 15 s), annealing (55 °C for 1 min) and extension (60 °C for 1 min). A final extension step at 60 °C for 30 s was also included. Negative qPCR controls containing no DNA were included to check for cross-contamination. qPCR standards were included on every plate to ensure consistency and accurate quantification across runs. A total of 9 plates were analyzed, with the qPCR reactions performed in triplicate for each sample and control. The standard curve for *B. edulis* mycelium quantification fulfilled the requirements for qPCR in terms of efficiency (R2 = 0.98; Efficiency = 96.9%) (Figure S1).

Quantification of *B. reticulatus* mycelium was achieved by targeting the ITS genomic region with a specifically designed primer pair: Bret_ITS2_F: 5′ GGTGAATCGCTTCCAATTCC 3′ and Bret_ITS2_R: 5′ GTCTCTCGAAGGTCAAAGGT 3′. To verify the amplification of the target region and generate the standard curve for qPCR, an endpoint PCR was first performed. This PCR amplified the ITS region from *B. reticulatus* DNA extracted from molecularly identified sporocarps. The PCR endpoint reactions (final volume of 12.5 μL) contain 1.25 μL (10 ng) of 1:10 diluted template DNA, 0.9 μM of the primer, 6.25 μL of Supreme NZYTaq 2x Green Master Mix (NZYTech), and ultrapure water (up to 12.5 μL). The thermal cycling conditions included an initial denaturation at 95 °C for 10 min, followed by 40 cycles of 95 °C for 15 s and 64 °C for 1 min. The PCR product was purified and sequenced to confirm amplification of the targeted ITS region of *B. reticulatus*. This verified amplicon was then used to generate the standard curve in the subsequent qPCR experiment.

The qPCRs were carried out in a final volume of 20 μL, containing 10 μL of NZY qPCR Probe Master Mix ROX plus (NZYTech), 0.25 μM of a probe, 0.9 μM of each amplification primer, 2 μL of template DNA, and ultrapure water (up to 20 μL). The thermal cycling conditions included an initial incubation at 95 °C for 10 min, followed by 40 cycles of denaturation at 95 °C for 15 s and a combined annealing/extension step at 64 °C for 1 min. Negative controls containing no DNA were included in each qPCR round to check for cross-contamination. Five-fold dilution series (performed in triplicate) of known ITS copy numbers (from 1.9 × 10^2^ to 7.95 × 10^5^ in 100 µL of DNA solution) from identified *B. reticulatus* sporocarp samples were used to establish the standard curve. The standard curve exhibited a satisfactory efficiency (R2 = 0.98; Efficiency = 99.5%) (Figure S2).

### 2.5. Alpha Diversity Parameters

Alpha diversity, which refers to the species richness and evenness of a microbial community within a single sample, was utilized in this study to analyze the diversity of bacterial and fungal communities in the soil samples collected across the sampling months.

The Operational Taxonomic Units (OTUs) identified in a sample served as a basic measure of species richness within the bacterial and fungal communities. The Shannon–Wiener (H′) diversity index considers both the number of species present and their relative abundance in a sample, providing a comprehensive picture of community diversity compared to species richness alone. Higher Shannon–Wiener index values indicate greater overall diversity within a sample. The Chao1 index is a non-parametric richness estimator that takes into account the presence of singletons (OTUs observed only once) in the data. Chao1 provides a more accurate estimate of the total species richness within a community compared to observed richness alone, acknowledging that some species might be present at very low abundances and therefore rarely detected. Inverse Simpson’s diversity index is a measure of diversity in the habitat, which takes into account the number of species present, as well as their relative abundance, highlighting the importance of rare species.

The alpha rarefaction curve representation depicts the accumulation of OTUs with increasing sequencing depth (number of reads analyzed). An ideal alpha rarefaction curve plateaus towards the end, indicating that sufficient sequencing depth was achieved to capture most of the species diversity within the samples. Alpha rarefaction curves generated using the Chao1 index were used to assess whether the sequencing effort (number of reads used in the analysis) was sufficient for identifying OTUs in the samples. Read depth refers to the average number of times each sequenced fragment (read) is captured, thus a higher read depth suggests a more comprehensive sampling of the bacterial and fungal community in each sample.

All diversity indexes were calculated using the free software EstimateS 9.1.0. (https://www.gbif.org/tool/81319/estimates-tool; accessed on 1 January 2021).

### 2.6. Statistical Analysis

Prior to statistical analysis, the sequencing data underwent quality filtering to remove OTUs with less than 10 total sequences. This filtering step helps to minimize the influence of potential sequencing errors and low-abundance background noise on the analysis [53]. Internal tests also showed minimal impact of these OTUs on overall community composition.

One-way analysis of variance (ANOVA) was employed to assess significant differences in bacterial and fungal alpha-diversity (species richness and diversity) between the sampling months, as well as significant changes in the prevalence and concentration of *B. edulis* and *B. reticulatus*. Mycelium data were log (x + 1) transformed prior to ANOVA to satisfy the assumption of equal variance between groups. Shapiro–Wilk and Levene’s tests to verify ANOVA assumptions after log transformation, were performed. The Shapiro–Wilk test indicated normality (W = 0.965, *p* = 0.234), and Levene’s test confirmed homoscedasticity (F = 1.12, *p* = 0.345). Following a significant ANOVA result, pairwise comparisons between individual sampling months were performed using Fisher’s Least Significant Difference (LSD) test to identify specific groups with statistically different means. Benjamini–Hochberg correction to control for false discovery rate in our multiple comparisons following ANOVA with LSD, was used. After correction, significant differences remained consistent, confirming the robustness of our results (adjusted *p*-values < 0.05). The significance level for all statistical tests was set at α = 0.05.

The prevalence of *B. edulis* and *B. reticulatus* mycelia in each sampling month was calculated as the proportion of soil samples testing positive for the presence of their mycelia using the qPCR data (number of positive samples divided by the total number of samples per month). Spearman’s rank correlation analysis was used to evaluate potential correlations between the concentration of *B. edulis* and *B. reticulatus* mycelia (measured by qPCR) and the relative abundance of bacterial and fungal species (determined by sequencing data). The significance level was set at α = 0.05 and only correlations with *p*-values below this threshold were considered significant and discussed.

The existence of significant differences between climatic data (temperature and precipitation) in September, October, and November was assessed by one-way ANOVA, followed by LSD test to identify specific months with statistically different values. Spearman’s rank correlation analysis was employed to evaluate potential correlations between the concentration of *B. edulis* and *B. reticulatus* mycelia (measured by qPCR) and the climatic parameters (temperature and precipitation) for each sampling month. Spearman’s rank correlation is a non-parametric test suitable for assessing relationships between variables that may not be normally distributed. The statistical tests were performed with GenStat release 15.1 (VSN International, Hemel Hempstead, UK).

To compare the composition of bacterial and fungal communities across the sampling months, non-metric multidimensional scaling (NMDS) analysis was used. Prior to NMDS, the OTU table was Hellinger-transformed to down weight the influence of highly abundant taxa and improve the visualization of compositional differences between samples. For the bacterial community analysis using NMDS, genus-level identifications were employed. However, for the fungal community analysis, NMDS was performed using taxa identified at order, family, genus and species level. This distinction acknowledges the limitations of metabarcoding technology for accurate species-level identification of fungi. Fungal metabarcoding amplicons are typically longer than 550 base pairs, whereas the technology can only sequence shorter fragments of the genetic markers (around 550 bp or less) [54].

Analysis of similarities (ANOSIM) with a one-way crossed layout for bacterial and fungal communities, using transformed data, with 999 permutations, was used to test for differences in bacterial and fungal communities between sampling months. The ANOSIM test is a comparative measure of the degree of dissimilarity between groups, calculated from resemblances. R value is a ratio of the between groups variation to the within group variation. R = 1 implies that all replicates within groups are more similar to each other than between groups, while R ≅ 0 implies little or no differences within and between the groups [55].

The SIMPER (Percentage Similarity Analysis) submodule was used in conjunction with ANOSIM to identify the specific taxa contributing most to the observed differences in community composition between sampling months. For Bray–Curtis similarities, SIMPER calculates the contribution of each taxon to the average Bray–Curtis dissimilarity between pairs of groups and within a group. A cumulative percentage cut-off of 70% was used to exclude the contribution of rare taxa to the overall dissimilarity. The significance level for all statistical tests was set at α = 0.05.

The software PRIMER 7.0.21 with the PERMANOVA+1 add was used for NMDS, ANOSIM, and SIMPER analyses.

## 3. Results

### 3.1. Soil Characterization and Climatic Conditions

The soils of the experimental stands were characterized by a sandy loam in texture, with a low bulk density (BD, 675 kg m^−3^) and high total porosity (Pt, 73%). However, the soil exhibited strong acidity (pH 4.6) with high organic carbon content (C, 6.9%) and a wide carbon to nitrogen ratio (C/N, 14). The cation exchange capacity (CEC) was weak (5.7 cmol(+) kg^−1^), with low base saturation (V, 31%) and very low levels of assimilable phosphorus (P, 1.7 mg kg^−1^) (Table S1).

The comparison of climatic parameters during the three sampling months revealed a decreasing trend in temperature (mean, maximum, and minimum) and potential evapotranspiration (PET) from September to November (Table S2). In contrast, October experienced a significant increase in precipitation (P) and water balance (WB) compared to the other sampling months (Table S2).

### 3.2. Bacterial Community

After quality filtering a total of 299,223 sequences (from 1,121,679 raw read depth), based on 16S rDNA, were generated. Analysis of the sequences revealed a total of 7778 OTUs defined at a 98–100% sequence similarity level. Nearly all OTUs (99.9%) were classified withing the bacterial domain, with a very small fraction (0.1%) being classified within the Archaea domain (Table S3). These OTUs were further classified into 18 phyla, 42 classes, 86 orders, 155 families, and 358 genera. Details on total read counts, OTU richness, and diversity indices (Chao1, Shannon–Wiener, Inverse Simpson index) for each sample are provided in Table S3. The rarefaction curve (Figure S3) plateaus towards the end, indicating that the sequencing depth utilized was sufficient to capture most of the bacterial diversity within the samples.

**Figure 1:**
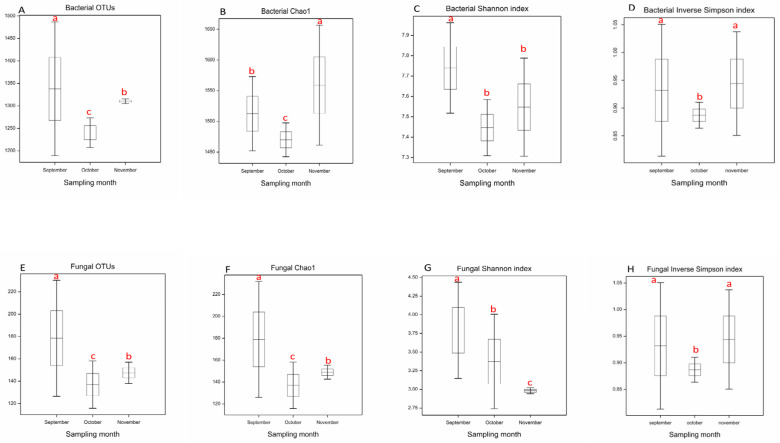
Bacterial (**A**–**D**) and fungal (**E**–**H**) alpha-diversity parameters over the sampling months. OTUs number and Chao1 index indicate species richness. Shannon–Wiener index, and Inverse Simpson diversity index show microbial diversity. The upper and lower edges of the boxes indicate the 75th and the 25th percentiles, respectively, whereas the upper and lower whiskers represent the maximum and minimum values. Different lowercase letters above the boxes represent significant differences between the groups after one-way ANOVA (α = 0.05).

Potential changes in alpha-diversity of the bacterial communities were assessed by comparing observed richness (OTU number), Chao1 estimator, Shannon–Wiener diversity index, and Inverse Simpson diversity index between sampling months (Figure 1). The highest OTU richness was observed in September, followed by a significant decrease in October and a partial recovery in November (*p* = 0.035) (Figure 1A). Chao1 showed a similar trend, with the highest value achieved in November (*p* = 0.021) after the decrease in October (Figure 1B). The Shannon–Wiener diversity index exhibited the highest value in September and remained relatively stable at lower values in October and November (*p* = 0.045) (Figure 1C). Inverse Simpson diversity index, showed highest values in September and November and a significant decrease in October (*p* = 0.041) (Figure 1D).

**Figure 2:**
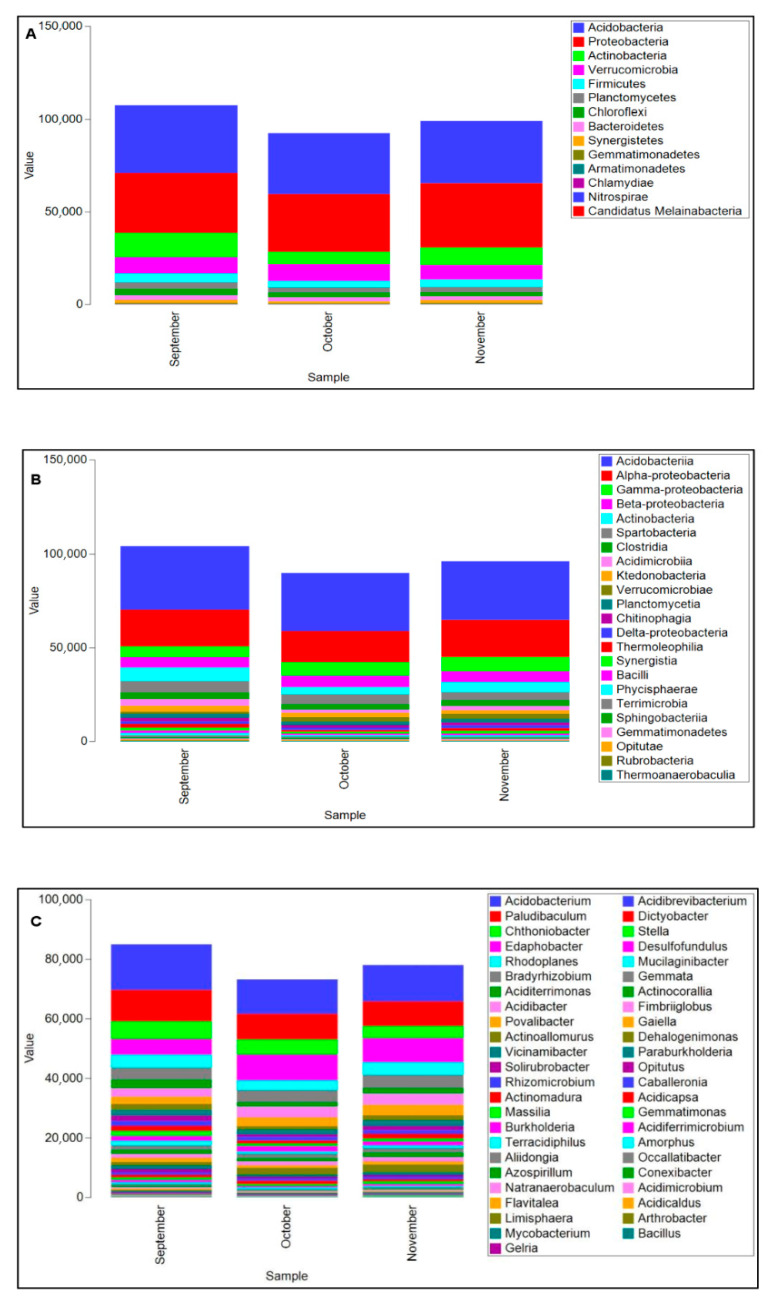
Relative abundance and assemblages of bacterial phyla (**A**), classes (**B**) and genera (**C**) inferred on 16S rRNA gene sequences, over the sampling months.

The dominant bacterial phyla across all samples were *Acidobacteria* (34% of the total sequences) and *Proteobacteria* (33%), followed by *Actinobacteria* (10%), *Verrucomicrobia* (9%), *Firmicutes* (4%), *Planctomycetes* (3%), *Chloroflexi* (3%), *Bacteroidetes* (2%), and *Synergistetes* (1%) (Figure 2A). The phyla *Gemmatimonadetes*, *Armatimonadetes*, *Chlamydiae*, *Nitrospirae* and *Candidatus Melainabacteria* were each present at less than 1% relative abundance.

At the class level, *Acidobacteria* (32%) and *Alpha-proteobacteria* (19%) were the most abundant, followed by *Gamma-proteobacteria* (7%), *Beta-proteobacteria* (6%), *Actinobacteria* (6%) and *Spartobacteria* (5%). All other classes were present at ≤3% relative abundance (Figure 2B).

*Acidobacterium* (12%), *Paludibaculum* (9%), and *Edaphobacter* (7%), all belonging to *Acidobacteria* phylum, were identified as the most abundant bacterial genera across all samples. Within the *Proteobacteria* phylum, *Bradyrhizobium* (8%) and *Rhodoplanes* (8%) (both *Alpha-proteobacteria*) and *Acidibacter* (4%) (*Gamma-proteobacteria*) were most abundant (Figure 2C). The relative abundance of *Rhodoplanes* and *Acidibacter* was significantly higher in September and in November compared to October (*p* = 0.025) as depicted in Figure 2C.

The most prominent genera inside *Actinobacteria* phylum were *Aciditerrimonas* (2% of the total sequences), *Actinoallomurus* (3%) and *Solirubrobacter* (3%). All three exhibited significant changes dependent on the sampling month, with *Aciditerrimonas* (*p* = 0.034), *Solirubrobacter* (*p* = 0.023) and *Actinoallomurus* (*p* = 0.011) being more abundant in September (Figure 2C). Similarly, the sequence abundance of *Chthonidiobacter* (*Verrucomicrobia*) significantly increased in September (*p* = 0.021) as illustrated in Figure 2C.

**Figure 3:**
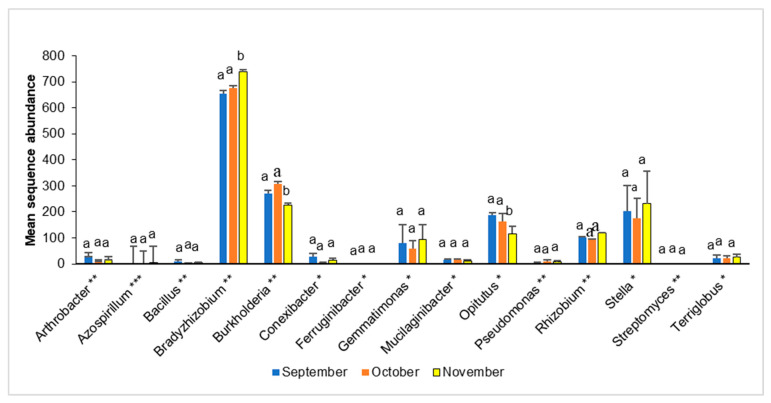
Mean sequence abundance (± SD) of bacterial genera considered indicators of productive sites of *B. edulis* sporocarps by [26] (marked with *), and of mycorrhiza helper bacteria by [33] (marked with **). The genus *Azospirillum* (***) has been cited by both authors. Columns marked with different lowercase letters were significantly different (α = 0.05) between the experimental plots by one-way ANOVA.

Fifteen bacterial taxa, considered as potential MHB involved in *B. edulis* complex development, were detected in the present study (Figure 3). Of these, the genera *Bradyrhizobium*, *Burkholderia* and *Opitutus* exhibited a significant shift in their mean sequence abundance across the sampling month (Figure 3).

**Figure 4:**
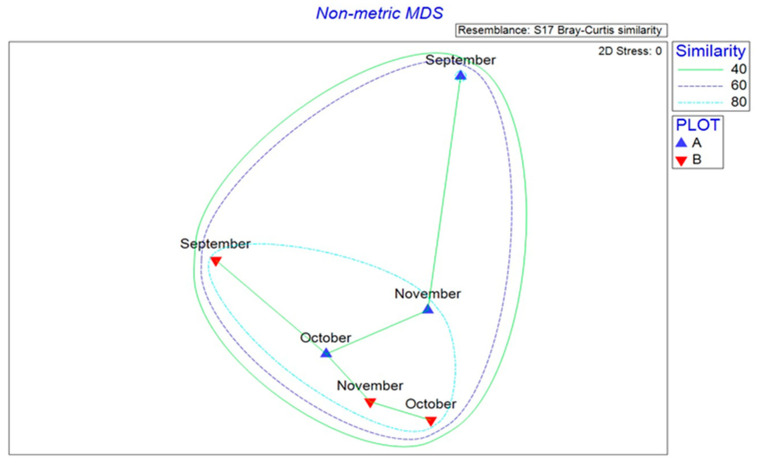
Two-dimensional non-metric multidimensional scaling (NMDS) ordination depicting the similarity between bacterial genera collected over the months. Minimum spanning tree visualizing the connections between similar bacterial assemblages is also shown. Letters A and B refers to the sampled stands.

The NMDS analysis yielded a two-dimensional ordination of the samples with a final stress value of 0.00, demonstrating the separation of the bacterial genera between September and the other two months (October and November) for both A and B experimental stands (Figure 4).

ANOSIM analysis detected the existence of significant difference in bacterial taxa assemblages between September/October (R = 0.65, *p* = 0.046), whereas the differences between September/November (R = 0.25, *p* = 0.058) and October/November (R = 0.5, *p* = 0.076) were not found to be significant.

SIMPER analysis revealed that the major contribution to those dissimilarities, in both September/October and September/November comparisons, was attributed to *Edaphobacter*, whereas in October/November the dissimilarity was primarily due to *Chthoniobacter* (Table 1).

### 3.3. Fungal Community

Illumina sequencing of the ITS2 region generated a total of 475,599 high-quality sequences, after quality filtering, from a raw read depth of 1,184,229 across the samples. Analysis revealed a total of 926 fungal OTUs classified into 8 phyla, 21 classes, 51 orders, 86 families, 99 genera, and 55 species. While only 37 OTUs remained unidentified at the phylum level, species-level identification was not achieved for 48% of the sequences. Details on total read counts, OTU richness, and diversity indices (Chao1, Shannon–Wiener, Inverse Simpson diversity index) for the fungal community are provided in Table S3. The rarefaction curve (Figure S4) plateaus towards the end, indicating that the sequencing depth utilized was sufficient to capture most of the fungal diversity within the samples.

Similar to the bacterial communities, variations in fungal alpha-diversity were assessed by comparing observed richness (OTU number), Chao1 estimator, and Shannon–Wiener, and Inverse Simpson diversity index, across the sampling months (Figure 1E–H). September exhibited the highest fungal diversity in terms of OTU richness (*p* = 0.021) (Figure 1E) and Chao1 index (*p* = 0.015) (Figure 1F). October displayed the lowest OTU richness and Chao1 values, while November had the lowest Shannon–Wiener diversity index (Figure 1G). On the other hand, Inverse Simpson diversity index showed the highest values in September and November, and a significant decrease in October ((*p* = 0.031) (Figure 1H).

**Figure 5:**
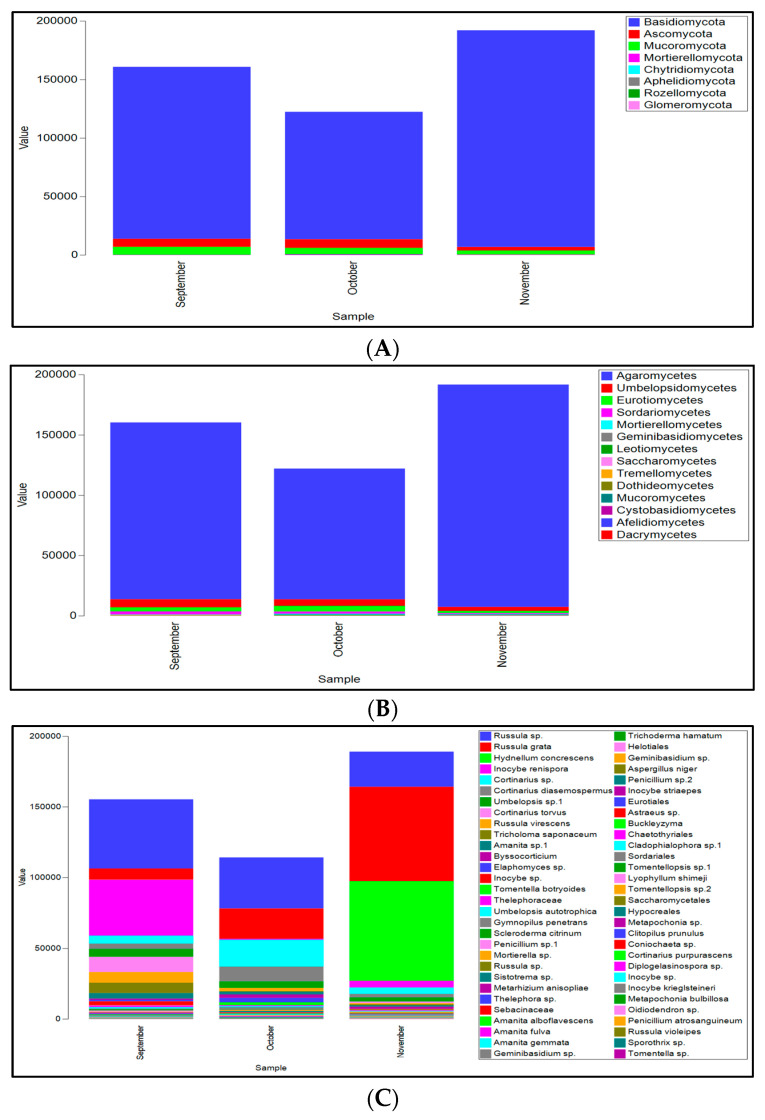
Relative abundance and assemblages of the most important fungal phyla (**A**), classes (**B**) and species (**C**) inferred on ITS rDNA gene sequences, over the sampling months. When OTUs were non identified at species level, order, family or genus are provided.

Analysis of dominant fungal taxa revealed that Basidiomycota was the most abundant phylum, representing 92% of the total fungal sequence reads (Figure 5A). Ascomycota and Mucoromycota followed in abundance at 4% and 3%, respectively. The remaining phyla (Mortierellomycota, Chytridiomycota, Aphelidiomycota, and Glomeromycota) were all present at less than 1% relative abundance (Figure 5A).

Basidiomycota was dominated by Agaromycetes class (93% of the total sequences), that also was the most abundant group in all the sampling months (Figure 5B). The genus Russula was the most abundant taxon (46% of the total sequences), represented by *Russula* sp. (23.4%), *R. grata* (20%), *R. virescens* (2.3%), *R. violeipes* (0.02%), *R. cyanoxantha* (0.02%), and a rare presence of *R. densifolia* (0.002%) and *R. parazurea* (0.002%). Other highly represented species belonging to this phylum were *Hydnellum concrescens* (15%), *Inocybe renispora* (9.4%), *Cortinarius* sp. (6.1%), *C. diasemospermus* (4%), *C. torvus* (2.5%), and *Tricholoma saponaceum* (1.8%), whereas the other species were below 1% (Figure 5C).

Variations in fungal species abundance across sampling months were observed (Figure 5C). *Russula* sp. (*p* = 0.028), *R. grata* (*p* = 0.040) and *H. concrescens* (*p* = 0.045) all significantly increased in November, whereas *Inocybe renispora* was dominant in September (*p* = 0.004) (Figure 5C).

Within the Ascomycota phylum, the class Eurotiomycetes reached 56% of the total sequence abundance (Figure 5B). The genus *Elaphomyces* (25%) was the most represented taxon within this class (Figure 5C).

The phylum Mortierellomycota was primarily represented by the genus *Mortierella*, being particularly abundant in October (*p* = 0.011). Mucoromycota phylum was mainly represented by the genus *Umbellopsis* (99% of the sequences within the phylum), and a marginal presence of *Gongronella* (1%). Interestingly, *Umbelopsis* sp. accounted for 2.7% of the total fungal sequences (Figure 5C).

**Figure 6:**
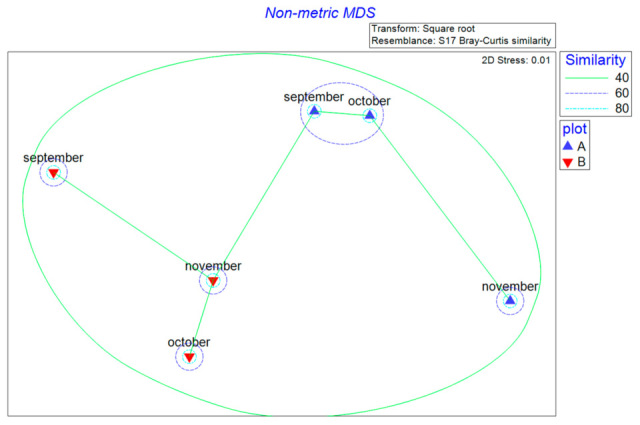
Two-dimensional non-metric multidimensional scaling (NMDS) ordination depicting the similarity between fungal genera collected over the months. Minimum spanning tree visualizing the connections between similar fungal assemblages is also shown. Letters A and B refer to the sampled stands.

NMDS analysis was performed to visualize the differences in fungal community composition between samples. The resulting two-dimensional ordination had a stress value of 0.01, indicating a reliable representation of the dissimilarities between samples (Figure 6). In experimental stand A, the fungal communities in September and October appeared similar (60% dissimilarity), whereas the November community exhibited a different composition compared to September and October. In contrast, the fungal communities in stand B for all sampling months (September, October, November) appeared distinct from those in stand A and from each other.

ANOSIM analysis statistically confirmed these observations. Significant differences in fungal assemblage composition were only detected between September and November (R = 0.78, *p* = 0.029). The differences between September/October (R = 0.28, *p* = 0.068) and October/November (R = 0.9, *p* = 0.077) were not statistically significant.

SIMPER analysis identified the key taxa contributing most to the dissimilarities between fungal communities (Table 1). For the September/October comparison, *Inocybe renispora* was the main contributor (49.65% dissimilarity). Between September and November, the major contributor was *Hydnellum concrescens* (54.46%). Finally, *H. concrescens* also played a dominant role in the dissimilarity between October and November (75.45%).

### 3.4. Boletus edulis and B. reticulatus Mycelium Prevalence and Concentration

By using qPCR, *B. edulis* mycelium was detected in 97% and *B. reticulatus* in 77% of the soil samples (Table 2). Mycelium prevalence did not change across the months for *B. edulis* (*p* = 0.234) or *B. reticulatus* (*p* = 0.112). However, *B. edulis* mycelium concentration (2.07 × 10^−6^ mg mycelium g^−1^ soil) was significantly higher than *B. reticulatus* (3.93 × 10^−8^ mg mycelium g^−1^ soil) (*p* < 0.001), and increased in November (*p* = 0.005), whereas *B. reticulatus* mycelium concentration peaked in October (*p* = 0.005) (Table 2).

The concentration of *B. edulis* mycelium appeared to be positively correlated with air maximum temperature, whereas *B. reticulatus* mycelium did not significantly correlate with any climatic parameter (Table 3).

### 3.5. Correlations Between B. edulis and B. reticulatus Mycelium Concentration and Soil Microbiota

A total of 81 bacterial species (only taxa above 1000 reads were considered) belonging to fifteen phyla were tested for the existence of significant correlations with *B. edulis* and *B. reticulatus* mycelium concentration (Table 4). Significant correlations were found in 30% of the cases with *B. edulis* and in 38% with *B. reticulatus*, with bacterial taxa from all the phyla, except for those belonging to *Nitrospirae*, *Synergistetes* and *Candidatus Melainabacteria*.

Mycelium concentration of *B. edulis* and *B. reticulatus* was positively correlated with six and eight bacterial genera, respectively (Table 4). *Edaphobacter* (*Acidobacteria*), *Singulisphaera* (*Planctomycetes*), *Massilia* (*Proteobacteria*), and *Lacunisphaera* (*Verrucomicrobia*) were involved in positive correlations with both Boletus species. The genus *Nitrosospherae* (*Thaumarcaheota*) exhibited positive correlations only with *B. edulis*, whereas *Mucilaginibacter* (*Bacteroidetes*), *Thermomicrobium* (*Chloroflexi*), and *Risungbinella* (*Firmicutes*) were positively associated only with *B. reticulatus*. Among potential MHB species, only *Rhizobium* (*Proteobacteria*), had a positive correlation with both *B. edulis* and *B. reticulatus* mycelium concentration.

Regarding the fungal community, a total of 107 taxa (abundance above 1000 reads were considered) belonging to Ascomycota, Basidiomycota, Mortierellomycota and Mucoromycota phyla were examined for correlations with B. edulis and B. reticulatus mycelium concentration (Table 5). Significant correlations were found in 20% of the cases for *B. edulis*, and 11% for *B. reticulatus*, with positive correlations detected in seven and five cases, respectively (Table 5). Positive correlations for *B. edulis* were found with Ascomycota (*Talaromyces leycettanus*) and Basidiomycota (*Amanita gemmata*, *Clavaria* sp., *Inocybe* sp., *Scleroderma citrinum*, and *Suillus* sp.). *B. reticulatus* exhibited positive correlation with fungal taxa from Ascomycota (Elaphomyces and Sordariales order) and Basidiomycota (*Astraeus* and *Entoloma* genera). Finally, the genus *Mortierella*, (Mortierellomycota) showed a significant positive correlation with both *Boletus* species (Table 5).

## 4. Discussion

During autumn in the Northern Hemisphere, soil microbial communities are likely to exploit the increased availability of readily degradable resources, such as proteins, sugars, and starches, facilitated by higher precipitation and soil moisture [56]. Precipitation (P) and water balance (WB) were significantly higher in October at the study sites. Soil moisture strongly influences microbial communities by impacting soil texture, bulk density, and oxygen availability, all of which can significantly affect bacterial development [57]. Despite the apparently favorable conditions in October, both bacterial and fungal richness and diversity exhibited a significant decrease, accompanied by a shift in species composition. Remarkably, a previous study conducted in nearby chestnut orchards during the same autumn season reported a similar seasonal effect on fungal species richness but not on bacterial diversity [27]. This contrasting observation suggests the possibility of distinct soil bacterial communities and litter decomposition dynamics in pedunculate oak stands when compared to chestnut orchards. Plant litter decomposition rates are known to be influenced by climatic factors, the surrounding microenvironment, litter chemistry, and the composition of soil microbiota and microfauna [58]. For instance, *Q. robur* litter is known to contain a higher proportion of recalcitrant material due to the presence of leaves with elevated calcium concentrations. These leaves not only decompose more slowly due to the inhibitory effects of calcium on ligninolysis but also contribute to a larger fraction of non-decomposable litter mass [59]. Consequently, oak litter likely has a higher lignin content compared to chestnut litter, and lignin concentration is well-established to have a strong negative correlation with litter decay rate [60].

Alonso Ponce et al. [61] developed models to characterize the realized niche of *B. edulis* in *Cistus ladanifer* scrubland. These models identified a preference for acidic soils with sandy loam texture, a high C/N ratio, low nitrogen availability, and limited concentrations of phosphorus, potassium, magnesium, and calcium. Similarly, Martínez-Peña et al. [22], confirmed the importance of a high C/N relation, low mineral nutrient availability and low pH for *B. edulis* associated with *P. sylvestris*. They additionally reported a positive correlation with sand content and water retention capacity. The soils characteristics of the experimental stands align closely with these reported preferences for *Boletus* species. The pedunculate oak soils exhibited high sand content, with a high porosity and a low bulk density. Also, these soils are very acidic, possess high total carbon content, a high C/N ratio and consequently have poor nutrient contents balance. All these soil characteristics of the oak stands thus represent an optimal habitat for *Boletus* species.

### 4.1. Bacterial Community Structure and Correlations with B. edulis and B. reticulatus Mycelium Concentration

The mean alpha-diversity parameters (species richness, Chao1 richness, Shannon–Wiener and Inverse Simpson diversity index) of bacterial community exhibited intra-seasonal fluctuations. These parameters reached the highest values in September, followed by a slight but significant decrease in October and a partial recovery in November. *Acidobacteria* and *Proteobacteria* were the dominant phyla in pedunculate oak stands soil community, agreeing with the results obtained in other habitats such as *Cistus ladanifer* scrubland [26], chestnut orchards [27], and *Eucalyptus* plantations [62], being differently affected by anthropogenic land-use alterations [63]. This finding suggests potential selection pressure for these bacterial taxa in habitats associated with ectomycorrhizal fungi.

The acidic characteristic of the *Q. robur* stand soils, a well-known driver for bacterial development, likely favors *Alpha-proteobacteria* and *Acidobacteria* species [64]. These bacterial groups are commonly associated with plant roots and plant biomass. The mechanisms for exploring these acidic soils include the utilization of metabolic intermediates like sugars, sugar alcohols (e.g., mannitol, sorbitol, xylitol) and organic acids, which are abundant in root exudates. Additionally, *Acidobacteria* may contribute to the degradation of plant-derived biomass through the secretion of extracellular enzymes [65].

The *Acidobacteria* phylum dominated the bacterial community, with genera *Acidobacterium*, *Edaphobacter* and *Paludibaculum* being the most abundant. The genus *Edaphobacter*, typically associated with *Russula* fungi in Fagaceae (beech) habitats, is considered an indicator of *Russula* sporocarp production [66]. *Edaphobacter*, was the primary driver of bacterial community dissimilarity between September and October, and between September and November. It also exhibited positive correlation with the mycelium concentration of both *Boletus* sp. However, previous studies in chestnut orchards reported a negative correlation between *Edaphobacter* and *B. edulis* [27]. Similarly, *Acidobacterium* and *Paludibaculum* showed negative correlations with *B. edulis* mycelium concentration in this study, while displaying positive correlations in chestnut soils [27].

The shift in bacterial assemblages found in October and November was primarily attributed to *Chthoniobacter*, belonging to *Verrucomicrobia* phylum. This phylum is recognized for its environmental significance, particularly in forest soil compared to pastures [67]. Soil *Verrucomicrobia* predominantly consists of unculturable members, yet plays a crucial role in agriculture through plant growth promotion, protection against soil-borne disease, and complex chemicals degradation [67]. While *Chthoniobacter* itself did not exhibit significant correlations, *Lacunisphaera*, another *Verrucomicrobia* genus, showed positive correlations with both *Boletus* species.

The genus *Massilia* (*Proteobacteria*) showed positive correlations for both *B. edulis* and *B. reticulatus*. This bacterium is recognized as a major group that colonizes the rhizosphere of many plant species [68]. *Massilia* along with other mycorrhiza helper bacteria (MHB) like *Bacillus*, *Paenibacillus*, *Rhizobium*, *Sinorhizobium*, *Arthrobacter*, *Streptomyces*, *Pseudomonas*, and *Herbaspirillum*, have been reported within arbuscular mycorrhizal fungi (AMF) spores [69].

Several genera previously identified by Mediavilla et al. [26] as significant indicators for productive *B. edulis* sporocarps in *Cistus ladanifer* habitats, were also found in this study, including *Azospirillum*, *Conexibacter*, *Gemmatimonas*, *Mucilaginibacter*, *Opitutus*, *Stella* and *Terriglobulus*. However, only *Mucilaginibacter* (*Bacteroidetes*) showed a significant positive correlation with *B. reticulatus* mycelium concentration. This plant-growth-promoting bacterium is known for its production of phytohormones and enzymes, which enhance nutrient uptake and protect plants from pathogens [70]. Despite its positive influence on *B. reticulatus* in this study, *Mucilaginibacter* did not exhibit a significant correlation with *B. edulis* mycelium concentration in chestnut soils [27].

Previous studies in chestnut orchards reported significant positive correlations between mycorrhiza helper bacteria (MHB) and *B. edulis* mycelium concentration [27]. These MHB included *Arthrobacter*, *Bacillus*, *Bradyrhizobium*, *Burkholderia*, *Pseudomonas*, *Paenibacillus*, *Rhizobium* and *Streptomyces*. In the present investigation, *Arthrobacter* (*Actinobacteria*), exhibited significant but negative correlations with both *B. edulis* and *B. reticulatus* mycelium concentration, while *Rhizobium* (*Proteobacteria*) showed positive correlation with both *Boletus* species.

*Rhizobia* group, encompassing *Rhizobium*, *Mesorhizobium*, *Bradyrhizobium*, *Azorhizobium*, *Allorhizobium and Sinorhizobium*, is generally known for forming nitrogen-fixing nodules in legumes [71]. However, this genus also includes non-nodulating strains associated with ectomycorrhizal fungi like *Suillus*, *Laccaria*, *Tuber* and *Tylospora* [72]. The positive correlation observed between *Rhizobium* and both *Boletus species* here, suggests a potential role for *Rhizobium* in promoting plant-fungus symbiosis by stimulating *Boletus* mycelial extension, although further investigations are necessary to confirm this hypothesis.

The positive correlations observed between both *Boletus* species and *Singulisphaera*, a genus with the ubiquitous *Planctomycetes* phylum represent a novel finding. *Planctomycetes* play a crucial role in global carbon and nitrogen cyclic [70]. *Singulisphaera* is an aerobic, moderately acidophilic and mesophilic budding bacterium known to utilize a wide range of sugars and polysaccharides as growth substrates [73].

Ectomycorrhizal fungi are known to select specific bacterial communities within the mycorrhizosphere that enhance plant growth and health [32]. These bacterial pools include plant-growth promoters, nitrogen fixers, and antagonists against pathogens. Our findings, showing significant correlations between rhizobacterial taxa with *B. edulis* complex mycelium development in both pedunculate oak and chestnut soils, suggest the existence of a core bacterial community associated with *Boletus* sp. mycelium. The specific nature of these interactions appears to be influenced by the host plant species. These results support the hypothesis that fungus-specific bacterial communities exist along or near the mycelium surface [74].

### 4.2. Fungal Community Structure and Interaction with B. edulis and B. reticulatus Mycelium

The high proportion (48%) of fungal sequences unassigned at the species level reflects limitations in current reference databases and marker resolution. Many environmental fungi remain underrepresented, hindering precise classification. This highlights the need for improved fungal taxonomic resources to enhance species-level identification in future studies. A general increase in fungal activity during late autumn, often attributed to the presence of more abundant plant litter, has been reported [75]. This trend is typically observed with saprotrophic fungi, which reach peak abundance in autumn, while ectomycorrhizal taxa are more prevalent during summer months [76]. Interestingly, both OTU abundance (species richness) and diversity of the fungal community significantly decreased from September to November, revealing a contrasting pattern. This finding partially aligns with observations in chestnut habitats, where species richness followed a similar decreasing trend across the sampling months, although diversity remained stable [27].

The observed decrease in fungal OTUs in October, mirroring the pattern seen in the bacterial community, strengthens the hypothesis of less favorable soil conditions for microbial growth during this month in pedunculate oak stands. Furthermore, the decline in microbial richness and diversity suggests a potential shift towards adaptation and specialization within specific basidiomycete communities as the litter decomposes throughout the autumn season. Among the five *Ascomycota* genera showing significant correlations with *B. edulis* mycelium concentration, only *Talaromyces leycettanus* displayed a positive correlation. This fungus, belonging to the ubiquitous soil-dwelling family Trichocomaceae, is known for its adaptability to extreme environments and association with decaying organic matter [77]. While detected in chestnut soils, *T. leycettanus* did not exhibit a significant correlation with *B. edulis* mycelium development [27]. This contrasting result highlights, once more, the potential influence of habitat-specific factors on microbial interactions.

Throughout the study period, the fungal community in the oak habitat was dominated by Basidiomycota. Within this phylum, genera commonly associated with productive *B. edulis* sites, such as *Amanita*, *Cortinarius*, *Inocybe* and *Russula*, were frequently observed [20,21,78].

Fifteen basidiomycete fungal taxa exhibited significant correlations with *B. edulis* mycelium concentration. Only the genera *Amanita*, *Clavaria*, *Inocybe*, *Scleroderma* and *Suillus* displayed positive correlations. The positive correlation observed with *Amanita* is consistent with the hypothesis of a beneficial co-existence between *B. edulis* and this genus [79]. These authors reported regularly co-occurrence of *B. edulis* with *A. muscaria* and *A. excelsa* across diverse geographical locations, including England, Austria, Italy, Sweden, USA, and New Zealand. In some cases, these fungal species were even observed forming composite mycorrhiza with closely interwoven rhizomorphs and hyphae. Furthermore, Ambrosio and Zotti [21], identify *Amanita* as an indicator species for *B. edulis* sites in broadleaf forests using Indicator Species Analysis (ISA). These findings, collectively support the potential for a positive interaction between *Boletus edulis* complex and *Amanita* species.

The genus *Clavaria* typically comprises terrestrial saprotrophic fungi. However, some evidence suggests that *Clavaria* species may form ericoid mycorrhizae with *Rhododendron* plant species [80]. The positive correlation observed between *Clavaria* sp. and *B. edulis* mycelium clearly requires further investigation to elucidate the potential nature of their interaction. *Scleroderma* is generally recognized as an antagonist of *Boletus* mycorrhizae in the field settings [27,81]. Thus, this study represents the first reported positive correlation between *Scleroderma* and *B. edulis* mycelium concentration. Under natural conditions, it is possible that *Scleroderma luteus* and *B. edulis*, both belonging to the same order, share similar environmental requirements, such as high soil moisture, organic matter availability, and reduced canopy cover, potentially leading to synchronized sporocarp formation [60,82].

In contrast, *Suillus* species are known for their easy to cultivation and frequently used in both field [81] and laboratory studies [83,84]. This may explain their abundance in the fungal assemblages. *Cortinarius purpurascens*, although abundant, displayed a negative correlation with *B. edulis* mycelium concentration. This finding aligns with the observation in mature chestnut orchards [27], suggesting a potentially inhibitory role of C. *purpurascens* on *B. edulis* development.

This study also offers the first comprehensive analysis of fungal partner interactions with *B. reticulatus* in *Q. robur* habitat. Only two ascomycetes displayed significant positive correlations with *B. reticulatus* mycelium concentration: *Elaphomices* sp. and an unidentified member of Sordariales order.

*Elaphomyces* is a well-known ectomycorrhizal fungus, recognized as one of the most important European “deer-truffle” genera [85]. These fungi are typically described in temperate and subarctic forest ecosystems, particularly in acidic soils. The other positive correlations, belonging to the highly diverse Sordariales order, remain unidentified at the genus level. The Sordariales encompass a wide range of ecological trophic strategies within soil-borne, lignicolous (wood-inhabiting), herbicolous (plant-associated), and coprophilous (dung-associated) taxa, classified across seven families [86]. Further investigation is needed to identify the specific Sordariales member associated with *B. reticulatus* in this habitat and elucidate the nature of their interaction.

Only two basidiomycetes, genera *Astraeus* sp. and *Entoloma* sp., showed positive correlations with *B. reticulatus* mycelium concentration among the seven fungi identified. *Astraeus* is a saprotrophic fungus described in chestnut ecosystems, associated with the production of *B. edulis* and *B. aereus* [87]. While the ecological role of *Astraeus* in relation to *B. reticulatus* is unclear and requires further investigation, the presence of *Entoloma* sp. agrees with observations in chestnut habitats. Several studies have documented the co-occurrence of *Entoloma* with sporocarps of *B. edulis* [88,89]. Furthermore, Ambrosio and Zotti [21] identified this genus as a significant indicator species for *B. edulis* complex sites in *Castanea sativa* by using the Indicator Species Analysis (ISA). These findings suggest a potential positive association between *Entoloma* and other *Boletus* species such as *B. reticulatus.*

*Mortierella* sp., the unique representative genus of the phylum Mortierellomycota identified in this study, exhibited positive correlations with both *B. edulis* and *B. reticulatus* mycelium concentration. *Mortierella* is a ubiquitous filamentous fungus with saprotrophic and endophytic trophic strategies. It is commonly isolated from the forest litter and plays a key role in the decomposition of organic matter in agricultural soils [90]. Certain *Mortierella* strains belong to the group of plant growth-promoting fungi (PGPF) [91]. These PGPF strains colonize bulk soil, rhizosphere (root zone) and plants tissues, and are known to produce several plant growth regulators such as indole—3-acetic acid (IAA), gibberellic acid (GA), and ACC deaminase, which alter plant ethylene biosynthesis [91]. Due to their potential for promoting plant growth, nutrient uptake efficiency and stress tolerance, as well as reducing the need for chemical fertilizers and pesticides, *Mortierella* sp. are attracting increasing research interest [92]. *Mortierella* may also exert positive effects on the soil microbiota, supporting beneficial microbial communities and ultimately enhancing crop yields. The positive correlation between *Mortierella* sp. and *Boletus edulis* complex species represents the first reported association with a basidiomycete fungus.

### 4.3. B. edulis and B. reticulatus Mycelium Prevalence and Concentration

The extraradical mycelium represents the most dynamic component of the mycorrhizal symbiosis, acting as a sensitive indicator of how mycorrhizal fungi respond to a complex web of environmental (biotic and abiotic) factors [93]. The higher concentration of *B. edulis* mycelium displayed in November contrasts with the results described for this species in nearby chestnut orchards where the maximum mycelium concentration occurred in September under similar weather conditions [27]. Similarly, De la Varga et al. [24] reported a decrease in *B. edulis* mycelium concentration in pine forest during October, probably because the great part of the fungal resources is allocated towards sporocarp fructification, which commonly occurs from October to December in this ecosystem.

In Italy, it has been reported that *B. edulis* mycelia were almost undetectable beneath sporocarps in highly productive sites, with scarce and scattered distribution patterns within the soil [20]. This could be due to the long-distance exploration type characteristic of *Boletus* mycorrhizae. These fungi possess extraradical mycelia concentrated as rhizomorphs, exhibiting a high degree of spatial heterogeneity [94]. Such pattern could also explain the irregular distribution in the soil. In the present study, however, both *B. edulis* and *B. reticulatus* were homogeneously spread across the sampled soils, being detected by qPCR in the 97% and 77% of the soil samples, respectively. These results suggest a potentially distinct mycorrhizal colonization strategy employed by *B. edulis* and *B. reticulatus* in the studied pedunculated oak habitat compared to observations in other geographic locations or with different host tree species.

The analysis of correlations between *Boletus* species and soil microbiota did not provide a clear explanation for the observed increase in *B. edulis* mycelium in November. The bacterial (*Aciditerrimonas*, and *Rhodoplanes*) and fungal taxa (*Russula* sp., *Russula grata* and *Hydnellum concrescens*) displaying higher abundance in November were not positively correlated with *B. edulis* presence. Once more, these findings urged further studies into the specific environmental factors or microbial interactions that may be influencing *B. edulis* mycorrhizal dynamics in this pedunculate oak habitat. De la Varga et al. [24] reported a positive correlation between *B. edulis* and rainfall, although negatively with the temperature. Conversely, Parladé et al. [23] observed a negative correlation between *B. edulis* mycelium biomass and monthly precipitation, with no significant effect of temperature. While *B. edulis* mycelium concentration showed a positive correlation with maximum air temperature in October, no other significant correlations with other climatic parameters were detected here. However, the impact of abiotic requirements on *B. edulis* mycelium concentration has recently been investigated by Santolamazza-Carbone et al. [42], who showed that climate–soil interactions, through the water balance and water availability in the soil, together with certain soil physio-chemical properties, have been the main determinants of mycelial development in chestnut orchards. Specifically, it has been indicated that low soil CEC associated with high water excess one month before the sampling, triggered a higher mycelium concentration in November.

In contrast with *B. edulis*, *B. reticulatus* mycelium concentration was higher in October, coinciding with higher precipitations, water balance, and a higher abundance of the soil fungus *Mortierella* sp. which showed positive correlation with this *Boletus* species, suggesting a potential interaction with both fungi. The impact of water balance on *B. reticulatus* mycelium concentration has been previously highlighted, being crucial the interaction between a high-water deficit one month before the sampling and a high soil moisture [42]. This means that under dry weather and water stress conditions, *B. reticulatus* mycelium can develop successfully only if the soil has high water retention capacity. Moreover, this observed decline in diversity in October, mirroring patterns in bacterial communities, is consistent with environmental pressures influencing microbial growth as autumn progresses. While the exact mechanisms driving temporal shifts can vary, studies highlighting diverse seasonal dynamics in ECM communities [95] or, conversely, relative temporal stability in others [96], underscore the need for further comparative research across different forest ecosystems to elucidate generalizable patterns. Integrating these environmental factors into future studies will be essential for unraveling the specific drivers underlying *Boletus* sp. mycelium development across diverse habitats.

## 5. Conclusions

This study provides novel insights into the intricate seasonal dynamics of soil microbial communities and their associations with *Boletus edulis* and *B. reticulatus* mycelium abundance in *Quercus robur* forests. However, as the research was conducted in a single locality, during one year, caution should be exercised when generalizing the findings beyond this specific context.

The positive correlations observed between specific bacterial and fungal taxa and the mycelium of *B. edulis* and *B. reticulatus* highlight the existence of functionally relevant microbial consortia adapted to the acidic, sandy, high C/N soils of pedunculate oak stands. Taxa such as *Rhizobium*, *Massilia*, and *Mortierella*, with known roles in plant growth promotion, nutrient mobilization, and mycorrhiza facilitation, likely contribute to the maintenance of soil nutrient cycling and host tree health, indirectly supporting *Boletus* productivity. Seasonal declines in microbial richness and diversity despite favorable October soil moisture suggest that oak litter chemistry, characterized by high lignin and calcium content, may favor specialized decomposers over generalist saprotrophs, thereby shaping ectomycorrhizal dynamics. These seasonal microbial shifts, coupled with the distinct temporal responses of *B. edulis* and *B. reticulatus* to precipitation and microbial partners, have clear management implications. Maintaining high soil porosity, moisture retention capacity, and acidity, preserving intact litter layers, avoiding nutrient enrichment, and conserving co-occurring ectomycorrhizal partners, may help sustain the microbial networks underpinning *Boletus* habitats. Incorporating microbial community monitoring into forest management could provide an early indicator of habitat suitability and seasonal fruiting potential, offering a predictive tool for sustainable *Boletus* resource management. Nevertheless, the correlative nature of the links found between soil microbiota and *Boletus* species limits causal interpretations, highlighting the critical need for targeted functional assays and manipulative experiments to elucidate the precise mechanisms driving these interactions. Future research should integrate fine-scale environmental data and apply multi-omics approaches to unravel the functional roles of microbial associates in *Boletus* sp. mycelium development. Furthermore, the development of predictive models incorporating detailed soil characteristics and climatic variables would significantly improve understanding of the ecological requirements and productivity of economically and ecologically important ectomycorrhizal fungi like *Boletus* species.

## Figures and Tables

**Table 1:** SIMPER results for dissimilarity (%) of bacterial and fungal taxa between the experimental sampling months. Contribution for dissimilarity (Contrib.%) of the first six taxa is shown.

**Bacteria**					
September/October (22.02%)		September/November (20.06%)		October/November (14.13%)	
Species	Contrib.%	Species	Contrib.%	Species	Contrib.%
*Edaphobacter*	8.67	*Edaphobacter*	7.94	*Chthoniobacter*	5.59
*Acidobacterium*	7.61	*Acidobacterium*	6.80	*Bradyrhizobium*	5.10
*Chthoniobacter*	5.18	*Chthoniobacter*	6.28	*Paludibaculum*	4.40
*Paludibaculum*	4.80	*Paludibaculum*	5.37	*Acidibacter*	4.22
*Aciditerrimonas*	3.40	*Limisphaera*	3.55	*Acidobacterium*	3.70
*Bradyrhizobium*	3.19	*Povalibacter*	2.93	*Rhodoplanes*	3.26
**Fungi**					
September/October (49.65%)		September/November (54.46%)		October/November (52.53%)	
Species	Contrib.%	Species	Contrib.%	Species	Contrib.%
*Inocybe renispora*	9.46	*Hydnellum concrescens*	10.81	*Hydnellum concrescens*	12.69
*Russula grata*	6.83	*Russula grata*	10.64	*Russula grata*	11.35
*Cortinarius* sp.	5.04	*Inocybe renispora*	8.30	*Russula* sp.	8.22
*Cortinarius torvus*	4.94	*Russula* sp.	7.25	*Cortinarius* sp.	5.17
*Cortinarius diasemospermus*	4.72	*Cortinarius torvus*	4.33	*Cortinarius diasemospermus*	4.83
*Russula* sp.	4.16	*Russula virescens*	3.72	*Inocybe renispora*	3.43

**Table 2:** Detection of *B. edulis* and *B. reticulatus* mycelium concentration (mg mycelium/g soil) in soil samples by qPCR. Thirty soil samples have been analyzed (2 repetitions × 5 soil samples × 3 sampling dates) for each *Boletus* species.

		*Boletus edulis*			*Boletus reticulatus*		
Experimental Stand	Sampling Point	September	October	November	September	October	November
1	1	0	2.46 × 10^−6^	2.40 × 10^−6^	6.88 × 10^−9^	2.08 × 10^−7^	3.46 × 10^−8^
1	2	2.36 × 10^−7^	1.19 × 10^−6^	1.23 × 10^−6^	1.16 × 10^−9^	1.75 × 10^−8^	7.16 × 10^−9^
1	3	2.28 × 10^−8^	7.84 × 10^−7^	1.15 × 10^−6^	1.31 × 10^−9^	2.14 × 10^−8^	1.90 × 10^−9^
1	4	3.12 × 10^−8^	5.15 × 10^−7^	1.35 × 10^−7^	1.31 × 10^−9^	6.48 × 10^−8^	3.06 × 10^−10^
1	5	1.05 × 10^−7^	5.94 × 10^−7^	4.33 × 10^−7^	3.18 × 10^−10^	0	0
2	1	2.31 × 10^−7^	1.42 × 10^−6^	5.05 × 10^−6^	0	0	8.68 × 10^−8^
2	2	8.60 × 10^−7^	2.01 × 10^−6^	4.63 × 10^−6^	4.36 × 10^−8^	5.20 × 10^−9^	2.18 × 10^−10^
2	3	1.88 × 10^−7^	4.49 × 10^−6^	1.37 × 10^−5^	2.04 × 10^−9^	1.47 × 10^−10^	9.00 × 10^−9^
2	4	3.03 × 10^−7^	2.1 × 10^−6^	5.62 × 10^−6^	0	0	2.92 × 10^−10^
2	5	1.84 × 10^−8^	1.41 × 10^−6^	8.77 × 10^−6^	1.40 × 10^−9^	6.64 × 10^−7^	0
Mean value per month		1.99 × 10^−7^	1.69 × 10^−6^	4.31 × 10^−6^	5.80 × 10^−9^	9.81 × 10^−8^	1.40 × 10^−8^
Total mean value		*B. edulis*: 2.07 × 10^−6^			*B. reticulatus*: 3.93 × 10^−8^		

**Table 3:** Correlations calculated with Spearman correlation coefficient analysis (significance level * *p* < 0.05) between *B. edulis* and *B. reticulatus* mycelia concentration and climatic parameters in September, October and November 2020. RH = relative humidity, P = precipitations, PET = monthly potential evapotranspiration, WB = water balance.

*Boletus edulis*				*Boletus reticulatus*		
Parameters	September	October	November	September	October	November
Mean T (°C)	0.026	0.326	0.199	−0.035	0.236	0.051
Maximum T (°C)	0.186	0.414 *	0.324	0.010	0.171	−0.180
Minimum T (°C)	−0.023	0.207	0.274	−0.083	0.289	0.049
RH (%)	−0.052	−0.160	0.079	−0.022	−0.160	0.150
P (mm)	0.061	−0.281	0.007	0.016	0.029	0.123
PET (mm)	0.035	0.230	0.197	0.024	0.134	−0.041
WB (mm)	−0.101	−0.317	−0.036	0.104	0.009	0.083

**Table 4:** Correlations between *B. edulis* and *B. reticulatus* mycelia concentration and the sequence abundance of 81 bacterial taxa, calculated with Spearman correlation coefficient analysis (significance level * *p* < 0.05). Only species with more than 1000 reads have been considered. Names in bold marked with ^1^ show bacterial genera known as indicators of highly productive sites of *B. edulis* sporocarps [26] whereas those marked with ^2^ show those taxa considered as mycorrhizal helper bacteria (MHB) (Frey-Klett et al. 2007) [33]. The genus *Azospirillum* ^1–2^ has been cited by both authors.

Taxon	*Boletus edulis*	*Boletus reticulatus*
*Acidobacteria*		
*Acidobacterium*	−0.943 *	−0.771 *
*Edaphobacter*	0.771 *	0.600 *
*Occallatibacter*	0.200	−0.314
*Paludibaculum*	−0.771 *	−0.429
*Silvibacterium*	0.086	0.086
*Terracidiphilus*	−0.771 *	−0.771 *
***Terriglobus*** ^1^	−0.029	−0.493
*Vicinamibacter*	−0.429	−0.943 *
*Actinobacteria*		
*Aciditerrimonas*	−0.886 *	−0.886 *
*Actinoallomurus*	−0.257	−0.600 *
*Actinomadura*	−0.200	−0.543
*Actinocorallia*	−0.486	−0.829 *
***Arthrobacter*** ^2^	−0.771 *	−0.771 *
***Conexibacter*** ^1^	−0.600 *	−0.620 *
*Gaiella*	−0.657 *	−0.657 *
*Mycobacterium*	−0.543	−0.886 *
*Solirubrobacter*	−0.371	−0.714 *
*Streptacidiphilus*	−0.986 *	−0.725 *
*Armatimonadetes*		
*Fimbriimonas*	−0.943 *	−0.600 *
*Bacteroidetes*		
*Chryseolinea*	0.486	0.143
*Flavitalea*	−0.841 *	−0.754 *
***Mucilaginibacter*** ^1^	−0.143	0.771 *
*Puia*	−0.771 *	−0.943 *
*Candidatus Melainabacteria*		
*Vampirovibrio*	0.471	0.557
*Chlamydiae*		
*Candidatus Protochlamydia*	−0.371	−0.371
*Neochlamydia*	−0.543	−0.714 *
*Parachlamydia*	−0.516	0.213
*Simkania*	−0.551	−0.683 *
*Chloroflexi*		
*Dehalogenimonas*	−0.189	−0.109
*Dictyobacter*	−0.246	−0.572
*Ktedonobacter*	−0.714 *	−0.546
*Thermanaerothrix*	0.551	0.464
*Thermogemmatispora*	−0.143	−0.314
*Thermomarinilinea*	0.464	0.029
*Thermomicrobium*	−0.213	0.871 *
*Thermosporothrix*	−0.595	−0.771 *
*Firmicutes*		
***Bacillus*** ^2^	−0.232	−0.232
*Brockia*	0.257	0.429
*Desulfofundulus*	−0.714 *	−0.714 *
*Desulfotomaculum*	−0.657 *	−0.829 *
*Gelria*	−0.657 *	−0.314
*Natranaerobaculum*	0.371	0.200
*Risungbinella*	−0.771 *	1.000 *
*Thermanaeromonas*	0.200	0.371
*Thermoflavimicrobium*	−0.486	−0.143
*Gemmatimonadetes*		
***Gemmatimonas*** ^1^	0.429	0.086
*Gemmatirosa*	−0.429	−0.771 *
*Roseisolibacter*	−0.257	−0.600 *
*Nitrospirae*		
*Nitrospira*	−0.371	0.143
*Planctomycetes*		
*Aquisphaera*	−0.754 *	−0.928 *
*Fimbriiglobus*	−0.348	−0.696 *
*Gemmata*	0.429	0.257
*Gimesia*	−0.116	−0.377
*Limnoglobus*	0.429	0.429
*Singulisphaera*	0.771 *	0.600 *
*Tepidisphaera*	−0.829 *	−0.314
*Thermostilla*	0.086	0.086
*Zavarzinella*	−0.203	−0.116
*Proteobacteria*		
*Acidibacter*	0.086	−0.257
*Acidibrevibacterium*	−0.486	−0.829
*Aliidongia*	−0.314	−0.314
***Azospirillum*** ^1–2^	0.086	0.086
***Bradyrhizobium*** ^2^	0.429	0.257
***Burkholderia*** ^2^	−0.486	−0.129
*Massilia*	0.771 *	0.943 *
*Povalibacter*	0.143	−0.143
***Rhizobium*** ^2^	0.671 *	0.743 *
***Pseudomonas*** ^2^	0.543	0.543
*Rhizomicrobium*	−0.371	−0.371
*Rhodoplanes*	−0.257	−0.600 *
***Stella*** ^1^	0.086	0.086
*Synergistetes*		
*Thermanaerovibrio*	0.200	0.029
*Thaumarchaeota*		
*Nitrosopumilus*	−0.093	0.000
*Nitrososphaera*	0.928 *	0.580
*Verrucomicrobia*		
*Chthoniobacter*	0.257	−0.429
*Lacunisphaera*	0.714 *	0.886 *
*Limisphaera*	0.314	0.314
***Opitutus*** ^1^	0.257	0.257
*Terrimicrobium*	0.257	−0.429
Total significant correlations	24 (30%)	30 (38%)
Total positive significant correlations	6	8
Total negative significant correlations	18	22

**Table 5:** Correlations between *B. edulis* and *B. reticulatus* mycelium concentration and the sequence abundance of 107 fungal taxa, calculated with Spearman correlation coefficient analysis (significance level * *p* < 0.05). For Basidiomycota and Ascomycota only species with more than 1000 reads have been considered.

	*Boletus edulis*	*Boletus reticulatus*
Taxon	Ascomycota	
*Acidomelania panicicola*	−0.338	0.068
*Alternaria alternata*	0.068	−0.034
*Anthracobia melaloma*	−0.131	−0.655
*Unidentified Ascomycota*	−0.543	−0.886 *
*Aspergillus* sp.	−0.135	0.270
*Aspergillus fumigatus*	−0.414	−0.414
*Aspergillus niger*	0.486	0.314
*Cenococcum geophilum*	−0.621	−0.828 *
*Cladophialophora* sp.1	0.371	0.371
*Unidentified Chaetotriales*	−0.152	0.395
*Chalara* sp.	0.655	0.131
*Cladophialophora* sp.1	0.371	0.371
*Cladophialophora* sp.2	−0.600	−0.600
*Coniella* sp.	−0.698 *	−0.152
*Coniochaeta* sp.	−0.143	0.029
*Cyclaneusma minus*	−0.778 *	−0.372
*Cyphellophora* sp.	−0.131	−0.655
*Diaporthe* sp.	−0.393	−0.131
*Diplogelasinospora*	0.522	0.174
*Elaphomyces*	−0.086	0.600 *
*Eurotiales*	−0.371	−0.371
*Fusarium* sp.	−0.655	−0.371
*Unidentified Helotiales*	−0.143	0.200
*Unidentified Hypocreales*	0.116	0.203
*Meliniomyces* sp.	−0.030	0.152
*Metapochonia* sp.	0.551	0.464
*Metapochonia bulbillosa*	−0.314	−0.657
*Metarhizium anisopliae*	−0.551	−0.464
*Metarhizium flavoviride*	−0.338	0.068
*Oidiodendron* sp.	−0.371	−0.371
*Oidiodendron myxotrichoides*	−0.845 *	−0.439
*Parateratosphaeria*	−0.135	0.270
*Penicillium* sp.	−0.257	0.086
*Pezicula* sp.	−0.406	0.290
*Pyrenochaeta*	0.101	0.507
Unidentified Sordariales	0.759	0.941 *
*Sporothrix inflata*	−0.429	0.086
*Talaromyces leycettanus*	0.679 *	0.586
*Trichoderma hamatum*	−0.714 *	−0.543
	**Basidiomycota**	
Unidentified Agaricales	0.257	0.257
Unidentified Agaromycetes	−0.655	−0.393
*Amanita* sp.1	−0.257	0.257
*Amanita* sp.2	0.455	−0.372
*Amanita* sp.3	0.455	−0.091
*Amanita alboflavescens*	−0.371	−0.371
*Amanita ceciliae*	0.388	0.034
*Amanita fulva*	0.455	−0.091
*Amanita gemmata*	0.741 *	0.439
*Astraeus* sp.	0.393	0.655 *
*Buckleyzyma* sp.	−0.131	−0.655 *
*Byssocorticium* sp.	0.086	0.429
*Clavaria* sp.	0.655 *	0.131
*Clitopilus prunulus*	−0.655 *	−0.393
*Cortinarius* sp.	0.200	0.543
*Cortinarius croceus*	−0.507	−0.101
*Cortinarius diasemospermus*	0.087	0.522
*Cortinarius purpurascens*	−0.755 *	−0.393
*Cortinarius torvus*	0.152	0.334
*Cryptococcus* sp.	0.131	0.393
*Cutaneotrichosporon* sp.	−0.655 *	−0.393
*Entoloma* sp.	0.393	0.655 *
*Exobasidium maculosum*	0.655	−0.393
*Galerina* sp.	−0.131	−0.655 *
*Geminibasidium* sp.	0.314	−0.200
*Gymnopilus penetrans*	−0.304	0.101
*Hydnellum concrescens*	−0.372	−0.676 *
*Hygrophoropsis aurantiaca*	−0.655 *	−0.393
*Hypholoma* sp.	−0.135	0.270
*Inocybe* sp.1	0.338	0.034
*Inocybe* sp.2	0.845 *	0.541
*Inocybe krieglsteineri*	0.030	0.577
*Inocybe renispora*	0.273	−0.273
*Inocybe striaepes*	0.131	0.393
*Lyophyllum shimeji*	−0.439	−0.541
*Microstroma* sp.	0.101	0.507
*Mycena* sp.	−0.655 *	−0.393
*Pholiota highlandensis*	−0.131	−0.655 *
*Rhizopogon* sp.	−0.393	−0.131
*Russula* sp.1	0.200	−0.314
*Russula* sp.2	0.038	0.034
*Russula cyanoxantha*	0.638	0.638
*Russula densifolia*	0.655	0.131
*Russula grata*	0.143	−0.543
*Russula parazurea*	−0.655	−0.393
*Russula violeipes*	−0.131	−0.655
*Russula virescens*	−0.943 *	−0.429
*Sarcodon* sp.	−0.393	−0.131
*Scleroderma citrinum*	0.889 *	0.395
*Scleroderma polyrhizum*	−0.393	−0.131
*Scytinostroma*	−0.655 *	−0.393
*Unidentified Sebacinaceae*	0.638	0.638
*Sistotrema* sp.	−0.068	0.338
*Solicoccozyma* sp.	−0.655 *	−0.393
*Spencerozyma*	−0.213	−0.030
*Suillus* sp.	0.655 *	0.131
*Thelephora* sp.	−0.232	−0.145
Unidentified Thelephoraceae	0.273	0.273
*Tomentella* sp.	−0.395	−0.577
*Tomentella botryoides*	−0.600	−0.943 *
*Tomentellopsis* sp.	−0.200	−0.029
*Trichoderma hamatum*	−0.714	−0.543
*Tricholoma saponaceum*	−0.638 *	−0.551
*Trichosporon* sp.	−0.655 *	−0.393
*Xenasmatella* sp.	−0.030	0.152
	**Mortierellomycota**	
*Mortierella* sp.	0.714 *	0.714 *
	**Mucoromycota**	
*Umbellopsis* sp.	−0.086	0.257
*Gongronella* sp.	−0.516	0.030
Total significant correlations	21 (20%)	12 (11%)
Total positive significant correlations	7	5
Total negative significant correlations	14	7

## Data Availability

The original contributions presented in this study are included in the article/Supplementary Materials. Further inquiries can be directed to the corresponding author.

## Supplementary Materials

The following supporting information can be downloaded at: https://www.mdpi.com/article/10.3390/microorganisms13092196/s1; Figure S1: Standard curve *Boletus edulis*; Figure S2: Standard curve *Boletus reticulatus*; Figure S3: Alpha rarefaction curve bacteria; Figure S4: Alpha rarefaction curve fungi; Table S1: physical and chemical soil parameters; Table S2: climatic parameters; Table S3: OTUs general information.
