# Supplementary material for: Metabarcoding Unveils Seasonal Soil Microbiota Shifts and Their Influence on Boletus edulis and Boletus reticulatus Mycelium in Quercus robur Stands
Source: Microorganisms. 2025 Sep 19;13(9):2196. doi: 10.3390/microorganisms13092196 (PMC12472445; doi:10.3390/microorganisms13092196)
Supplement: Supplementary file 1 [file microorganisms-13-02196-s001.zip › microorganisms-3736747-supplementary.pdf]

Supplementary material

Metabarcoding Unveils Seasonal Soil Microbiota Shifts and Their Influence on *Boletus edulis* and *Boletus reticulatus* Mycelium in *Quercus robur* Stands

Serena Santolamazza-Carbone, Laura Iglesias-Bernabé, Elena Benito Rueda, Esther Barreal, Pedro Pablo Gallego

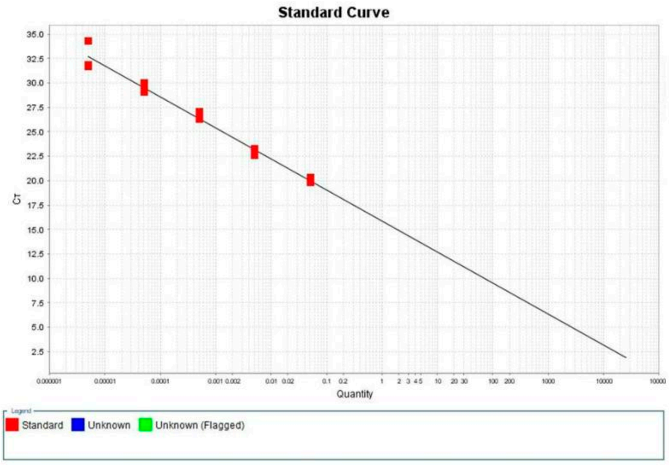

Figure S1. Standard curve obtained for mycelium quantification of *B. edulis* by qPCR. Curve was generated by plotting the Ct values against the logarithm of known amounts of mycelium. The efficiency of the qPCR (E) was calculated as E = (10−1/ slope−1) × 100.

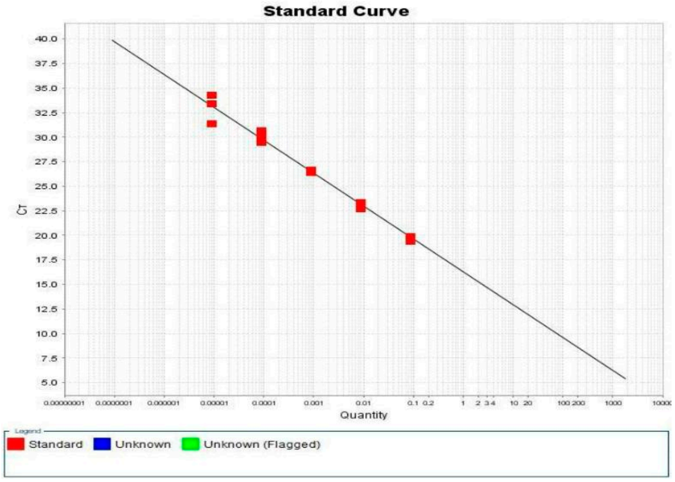

Figure S2. Standard curve obtained for mycelium quantification of *B. reticulatus* by qPCR. Curve was generated by plotting the Ct values against the logarithm of known amounts of mycelium. The efficiency of the qPCR (E) was calculated as E = (10−1/ slope−1) × 100.

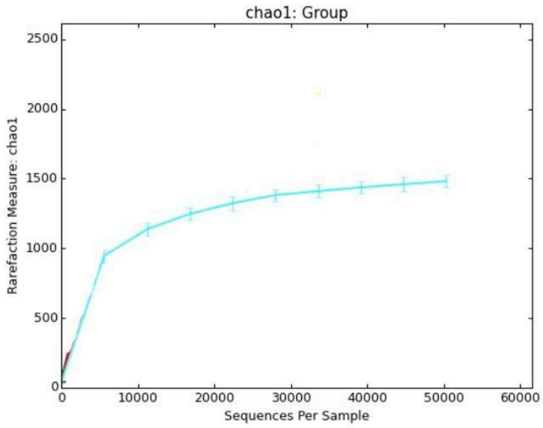

Figure S3. Alpha rarefaction curve (with Chao1 method), showing whether the number of reads used in the DNA metabarcoding analysis was sufficient in identifying bacterial OTUs by using the 16S rRNA gene sequence. Thirty samples have been studied (five samples x two repetition plot x 3 sampling dates).

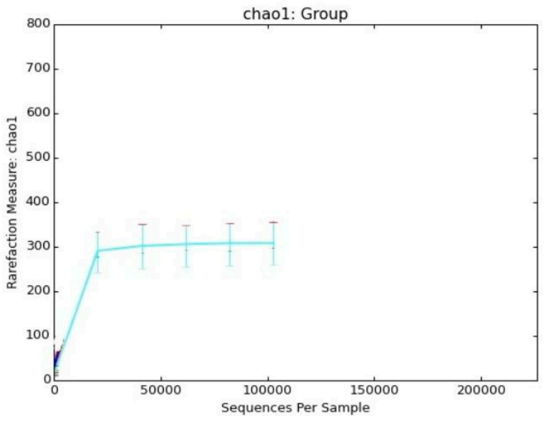

Figure S4. Alpha rarefaction curve (with Chao1 method), showing whether the number of reads used in the DNA metabarcoding analysis was sufficient in identifying fungal OTUs by using ITS region of rDNA gene sequences. Thirty samples have been studied (five samples x two repetition plot x 3 sampling dates).

Physical parameters	
Sand %	63.90±3.21
Silt %	18.72±2.32
Clay %	17.39±1.11
Soil texture	Sandy-loam
BD (kg/m3)	675.05±107.85
Pt (%)	72.77±4.19
**Chemical parameters**	
pH	4.60 ± 0.12
N %	0.48 ± 0.06
C %	6.89 ± 1.05
C:N	14 ± 1
P mg Kg^{-1}	1.71 ±0.78
Ca cmol(+)kg^{-1}	0.66 ± 0.20
K cmol(+)kg^{-1}	0.30 ± 0.02
Mg cmol(+)kg^{-1}	0.48 ± 0.05
Na cmol(+)kg^{-1}	0.41 ± 0.11
Al cmol(+/kg^{-1}	69.01± 7.41
CEC (cmol(+)kg^{-1}	5.68 ± 1.25
V %	30.99 ±7.41

Table S1. Physical and chemical soil parameters (mean ± SD) assessed from soil samples collected in September 2020. CEC = Cation Exchange Capacity, V = Base Saturation, BD = bulk density, Pt = total porosity.

Climatic parameters	September	October	November	P value
Mean T(ºC)	18.70 ± 0.47 a	14.46 ±0.34 b	12.96 ± 0.46 c	P<0.001
Maximum T(ºC)	25.03 ±0.77 a	18.88 ±0.35 b	12.96 ±0.37 c	P<0.001
Minimum T(ºC)	13.83 ±0.46 a	11.09 ± 0.46 b	9.15 ± 0.55 b	P<0.001
RH (%)	81.10 ±2.05 a	90.00± 1.10 b	90.77 ±1.10 b	P<0.001
P (mm)	59.20 b	173.30 a	105.80 b	P = 0.052
PET (mm)	103.30 a	53.70 b	38 b	P<0.001
WB (mm)	-39.40 c	121.50 a	67.60 b	P =0.031

Table S2. Climatic parameters of the sampling months obtained from the regional meteorological service (www.meteogalicia.gal) are shown. For P, PET and WB, the sum of the daily amount is provided. Within rows, results followed by the same letter are not significantly different by ANOVA (α = 0.05). RH = air relative humidity, P = precipitation, PET = potential evapotranspiration, WB = water balance.

			Bacteria			
Repetition plot	Sampling month	Total read bases	Raw read count	OTUs	Chao1	Shannon-Wiener index
1	September	86,453,828	192,700	1,408	1,541	7,844
1	October	87,067,328	193,226	1,256	1,483	7,512
1	November	87,761,861	195,096	1,308	1,513	7,661
2	September	74,059,392	164,856	1,268	1,484	7,635
2	October	79,823,853	177,529	1,225	1,457	7,382
2	November	89,209,494	198,272	1,313	1,605	7,434
			**Fungi**			
**Repetition plot**	**Sampling month**	**Total read bases**	**Raw read count**	**OTUs**	**Chao1**	**Shannon-Wiener index**
1	September	78,383,241	204,200	203	204	4,097
1	October	91,707,671	230,839	127	127	3,672
1	November	105,223,523	267,513	143	146	3,001
2	September	62,417,058	162,053	154	154	3,488
2	October	57,245,607	144,336	147	147	3,075
2	November	70.381.284	175.234	152	152	2,963

Table S3. Total read bases, read count and OTUs (species richness) relative to bacterial and fungal community.
